# Deep anterior lamellar keratoplasty following thermokeratoplasty assisted epikeratophkia: A novel two-stage one-graft method to treat acute corneal hydrops

**DOI:** 10.3389/fmed.2022.1080892

**Published:** 2023-01-12

**Authors:** Chunyu Liu, Xinyu Huang, Jiaqi Shen, Yushan Zhang, Li Zhang, Yanlong Bi

**Affiliations:** Department of Ophthalmology, School of Medicine, Shanghai Tongji Hospital, Tongji University, Shanghai, China

**Keywords:** acute corneal hydrops, epikeratophakia, deep anterior lamellar keratoplasty, anterior segment optical coherence tomography, Descemet membrane

## Abstract

**Purpose:**

To evaluate the clinical effects of deep anterior lamellar keratoplasty (DALK) using a single graft after thermokeratoplasty assisted epikeratophakia for the treatment of acute corneal hydrops.

**Methods:**

This novel surgical procedure was performed on seven eyes of seven patients between 2019 and 2020. The procedure combines a first-stage surgery of thermokeratoplasty assisted epikeratophkia with intracameral sterile air injection and a second-stage surgery of DALK using the same corneal graft for both procedures. Main outcome measures included pre- and postoperative corrected distance visual acuity (CDVA) and anterior segment optical coherence tomography (AS-OCT) parameters. Corneal transparency, epithelization, and the presence of neovascularization, were evaluated at the 1-year follow-up visit.

**Results:**

Corneal edema resolved rapidly in six of the seven cases. The mean central corneal thickness was significantly reduced from baseline to 1 day, 1 week, 1 month, and 2 months after the first-stage surgery (*P* < 0.0001). At a mean of 2.1 ± 0.7 months after the first-stage surgery, DALK was successfully performed in all cases. Six months later, the mean central corneal thickness was 611 ± 31 μm and the mean thickness of the recipient’s residual stroma bed was 20 ± 6 μm at the central corneal area. Mean LogMAR CDVA improved from 1.74 ± 0.34 at baseline to 0.20 ± 0.11 after DALK (*P* < 0.0001). No postoperative complications appeared in our case series during the 1-year observation period.

**Conclusion:**

Very good visual results were obtained with a novel technique (thermokeratoplasty assisted epikeratophakia followed by DALK using the same corneal graft) in the treatment of acute corneal hydrops.

## Introduction

Keratoconus is a chronic ectatic corneal disorder whose etiology, which seems to involve the inflammatory cascade, and is related to mechanical trauma from the habit of rubbing the eyes and nocturnal ocular compression (1, 2), is still the subject of extensive debate, is characterized by the presence of irregular astigmatism and corneal thinning, which usually progress during the first three decades of life (3, 4). Acute corneal hydrops (ACH), which may result in corneal scars and vision loss, is an uncommon complication of keratoconus secondary to rapid stromal edema, and even intrastromal clefts (pseudocysts) formation, secondary to Descemet membrane rupture (5, 6). ACH is usually self-limiting and generally resolves without intervention over 2–4 months (7), and may preset complications such as neovascularization (8), infection, malignant glaucoma, and corneal perforation (9, 10). Surgical interventions has been suggested in order to in order to accelerate the resolution of corneal edema. A history of ACH may also increase the risk of endothelial graft rejection after penetrating keratoplasty (11). Deep anterior lamellar keratoplasty (DALK) is now the preferred method to treat deep corneal stromal lesions. During DALK, the healthy endothelium of the recipient cornea can be preserved, avoiding postoperative endothelial immune rejection (12) and chronic endothelial dysfunction (13). The graft is cell-extracted and preserved by freezing in glycerol, so the risk of stromal immune rejection is also low after DALK (14). However, when ACH arises in keratoconus, disruption of Descemet’s membrane (DM) and the endothelium may create difficulties in performing a complete DALK (11, 15). In this study, we designed a novel and safe two-stage surgical procedure. The first stage involves thermokeratoplasty assisted epikeratophakia combined with intracameral air injection to accelerate the ACH healing and the second stage is the DALK procedure using the same graft.

## Materials and methods

### Ethical approval

This retrospective, interventional, non-randomized observational study was approved by the ethics committee of the Shanghai Tongji Hospital of Tongji University, Shanghai, China. After a thorough explanation about the nature of the study, all patients agreed to participate and provided written informed consents to participate prior to study entry. Signed consent was obtained from patients for all clinical photographs that permit their identification and is archived by the authors. Furthermore, this study conformed to the ethical standards outlined in the Declaration of Helsinki.

### Patients

In this study, the novel procedure was performed on seven eyes of seven patients between February 2019 and May 2021 at the department of ophthalmology affiliated with the Tongji University School of Medicine. The patients were aged between 17 and 21 years (mean 18.9 ± 1.4 years), and all were diagnosed with ACH immediately after onset of symptoms. Among the cases, the mean ACH duration was 25.9 ± 6.1 days. None of the patients had previously undergone corneal surgery or been treated with rigid gas permeable (RGP) contact lenses. All patients underwent relevant ophthalmic examinations pre and postoperatively, including slit-lamp examination and CDVA. The extent of corneal edema, corneal thickness, the location and size of the DM breaches were evaluated by anterior segment optical coherence tomography (AS-OCT) examination.

### Surgical procedure

**FIGURE 1:**
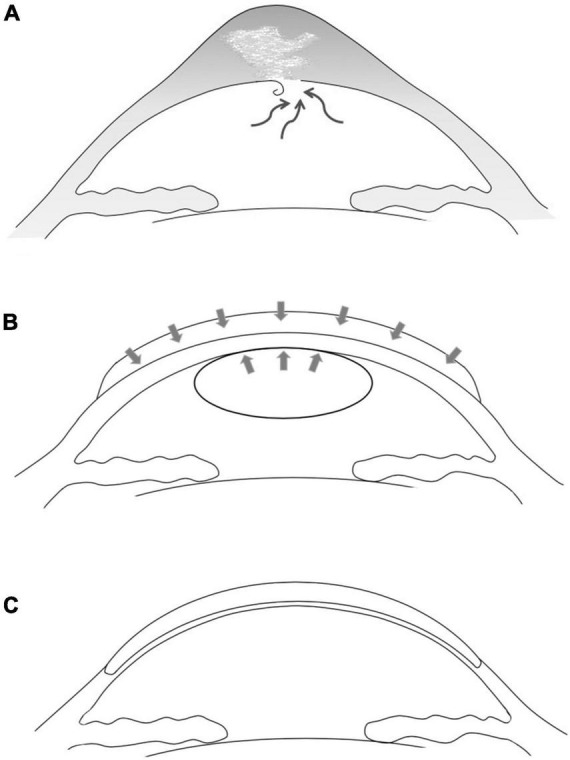
Schematic drawing of the two-stage one-graft method. **(A)** Acute corneal hydrops in advanced keratoconus due to rupture of Descemet’s membrane and subsequent intrastromal fluid accumulation. **(B)** First-stage surgery. The pathological cornea was covered and pressed with an allogenic donor graft and the detached DM was pushed upward by an injected sterile air bubble. The disrupted DM was allowed to reset and heal, and the severe edema subsided rapidly. **(C)** Approximately 6 months later, the second-stage DALK was performed using the same donor graft.

All surgeries were performed by the same surgeon (YL Bi). The first-stage surgery was thermokeratoplasty assisted epikeratophakia combined with injection of sterile air into the anterior chamber. The graft bed was prepared firstly, followed by cauterization of the edematous cornea using bipolar electrocautery forceps. During this procedure, the epithelium of the pathological cornea was cauterized and stripped, and the subepithelial tissue was also cauterized until the pathological cornea was visibly flatter. At this step, the heat of the cauterization was controlled within the appropriate range to minimize corneal stromal scarring and damage to the corneal endothelium. The corneal cone apex position was determined by slit lamp microscopy and corneal topography before surgery (the mean cone bottom diameter of this group of cases was 8.51 ± 0.26 mm). A partial thickness trephination was then made to a depth of about 200 μm with a vacuum trephine of corresponding dimension, and a peripheral pocket was created for insertion of the edge of the donor graft. The prepared donor graft was a decellularized homograft which had been stored at –78°C in pure sterile glycerin, and 1.5 mm larger than the previous trephine (the average of this group of cases is 10.07 ± 0.32 mm) was then sutured in place with interrupted 10-0 nylon. A paracentesis of the anterior chamber was made at the 9 o’clock position to decrease the intraocular pressure, and about 0.1 ml sterile air was then injected into the anterior chamber to compress the break in the pathological DM (Figures 1A, B). The eye under treatment was covered with a bandage contact lens and a compression bandage. The patient was instructed to rest in the supine position as much as possible after surgery, avoiding strenuous exercise and eye rubbing.

The second-stage surgery was DALK. About 2 months after the epikeratophakia, DALK was performed using the same corneal graft. The edge of the peripheral stromal pocket and the edge of the donor graft were identified and the donor graft was then completely dissociated and temporarily preserved in an empty moist chamber. After initial trephination, the incisal edge was held using fine toothed forceps and was dissected at about 4/5 thickness. During this process, the manual dissections were performed carefully three to five times in most cases to avoid further damaging the DM. The Anwar bubble technique should never be used on such patients. When the anterior stroma was dissected (at approximately 50% thickness), a sterile air bubble was injected into the anterior chamber to avoid rapid aqueous leakage from the reopening of the healed DM breaks and its further edema. If modest leakage was observed from the stromal bed, the manual dissection was conducted more carefully and quickly. In all of our cases, the same donor grafts were used in the second-stage DALK. The graft was reversed, placed, and trimmed on the cutting-off table using a trephine 0.5 mm larger than the recipient bed. During this procedure the corneal epithelium was carefully protected. The donor graft was then fixed using 10-0 interrupted nylon sutures (Figure 1C). effusion between graft and bed was observed after DALK. After topical anesthesia, a lacrimal irrigating needle was used to gently open the wound between the two sutures at 5 o’clock. The wound was pressed down gently to help the interlaminar fluid flowing out, the double anterior sign disappeared immediately.

### AS-OCT examination

**FIGURE 2:**
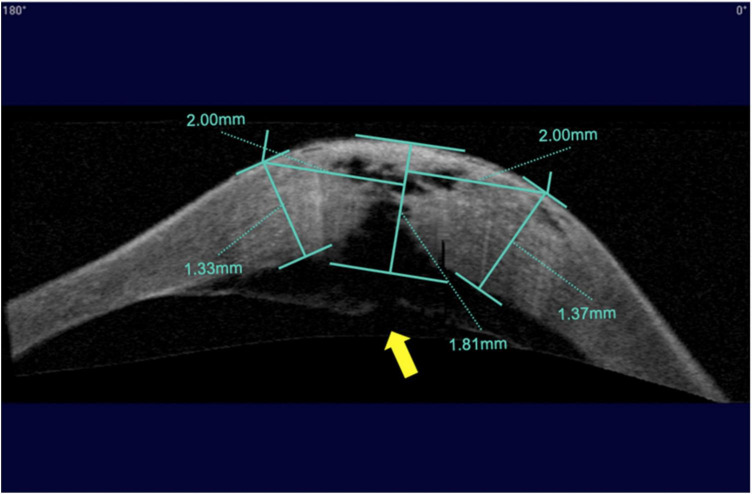
Measurement of corneal thickness using anterior segment optical coherence tomography (AS–OCT) after acute corneal hydrops. Indicated from left to right are the corneal thickness 2 mm nasal to the corneal apex with a horizontal scan, the corneal thickness at the apex, and the corneal thickness 2 mm temporal to the corneal apex with a horizontal scan. The yellow arrow indicates a break in Descemet’s membrane.

The corneal morphologic examination was performed using AS-OCT (Carl Zeiss Meditec, USA). Preoperative measurements included the thickness of the corneal apex (T_a_), the corneal thickness 2 mm nasal and temporal to the corneal apex, as determined by horizontal (T_nh_, T_th_) and vertical (T_nv_, T_tv_) scans (Figure 2). If the DM was detached, the width of the DM detachment was measured using different scan directions, and the maximum distance from the detached membrane to the posterior corneal surface was also determined. The above measurements and the thickness of the central recipient bed (Tr) were measured at 1 day, 1 week, 1 month, and 2 months after the first-stage surgery and again at 6 months after the second-stage surgery.

### Statistical analysis

Data were analyzed statistically using SPSS Version 21.0 (SPSS Inc., IBM, USA), and the results were expressed as mean ± standard deviation. After performed the first-stage surgery, one way ANOVA was used to test the T_a_, T_*r*_, T_nh_, T_th_, T_nv_, and T_tv_ values at preoperative and different time points after surgery (1 day, 1 week, 1 month and 2 month). Before and 6 months after DALK, a paired samples *t*-test was used to analyze the preoperative and postoperative T_a_, T_nh_, T_th_, T_nv_, and T_tv_ values, with *P* < 0.05 considered statistically significant.

## Results

**FIGURE 3:**
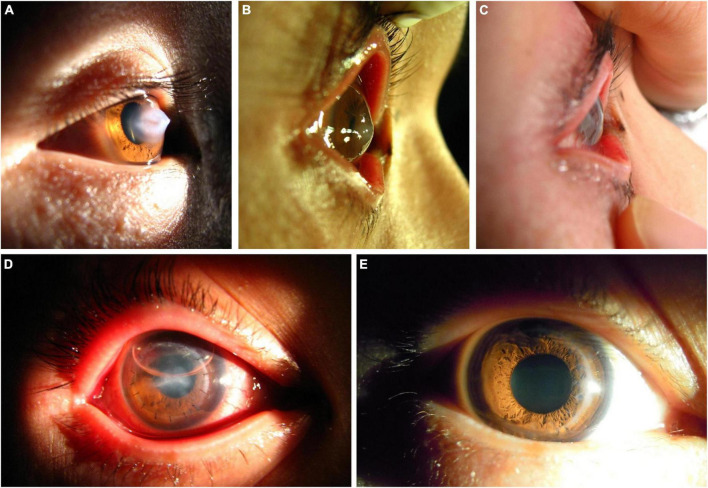
A patient with ACH treated with the epikeratophakia–DALK combination after a first treatment with thermokeratoplasty. **(A)** Acute onset ACH, with severe edema of the corneal stroma. **(B)** At 2 weeks after thermokeratoplasty a recurrence of edema was evident centrally, and epikeratophakia was performed immediately. **(C,D)** At 2 days after epikeratophakia, the corneal haze and edema had noticeably subsided. **(E)** At 3 months after DALK using the same graft, the cornea was transparent.

All seven surgeries were performed successfully without intraoperative or postoperative complications. After the first stage surgery, the DM was reattached to the posterior corneal stroma in all patients. In six patients, the corneal edema subsided rapidly 1 day after surgery and gradually decreased over the next 2 weeks (Table 1). Edema in one case took 5 days to resolve due to a large break in the DM (Figure 2). One patient, who had been treated with thermokeratoplasty for emergency management upon diagnosis of ACH had a recurrence of edema 2 weeks after surgery and had to undergo epikeratophakia to release the corneal hydrops (Figure 3).

**TABLE 1:** AS-OCT results preoperatively and 2 months after the first-stage surgery.

	Pre-operation	1 day	1 week	1 month	2 months
T_a_ (μm)	1,870 ± 243	1,373 ± 221*	1,186 ± 129*	1,157 ± 131*	1,063 ± 125*
T_r_ (μm)	/	778 ± 120	603 ± 72^#^	567 ± 21^#^	520 ± 10^#^
T_nh_ (μm)	1,368 ± 244	1,284 ± 216*	1,153 ± 154*	1,124 ± 142*	1,042 ± 97*
T_th_ (μm)	1,412 ± 263	1,307 ± 239*	1,172 ± 150*	1,136 ± 145*	1,050 ± 101*
T_nv_ (μm)	1,344 ± 269	1,221 ± 218*	1,130 ± 180*	1,104 ± 171*	1,033 ± 138*
T_tv_ (μm)	1,238 ± 258	1,189 ± 238*	1,118 ± 200*	1,080 ± 173*	1,021 ± 129*

Ta, corneal apex thickness; Tr, central recipient bed thickness; Tnh, corneal thickness 2 mm nasal to the corneal apex, determined with a horizontal scan; Tth, corneal thickness 2 mm temporal to the corneal apex, determined with a horizontal scan; Tnv, corneal thickness 2 mm nasal to the corneal apex, determined with a vertical scan; Ttv, corneal thickness 2 mm temporal to the corneal apex, determined with a vertical scan.

*P value < 0.05 compared with the preoperative values.

^#^*P* value < 0.05 compared with the values measured 1 day after surgery.

Before the second-stage surgery, all breaks in the DM were almost healed and the corneal stroma was in a non-edematous condition, based on the corneal morphologic parameters measured using AS-OCT. In this case series, the mean period was 2.1 ± 0.7 months from the completion of epikeratophakia to DALK. During the second stage DALK, the pre-DM corneal stroma was exposed as much as possible. In two cases, slow aqueous leakage was observed in the deep corneal stroma when the manual dissection was performed close to the DM. In addition, mild effusion between graft and bed was observed in one patient 1 day after DALK. After topical anesthesia, a lacrimal irrigating needle was used to gently open the wound between the two sutures at 5 o’clock. The wound was pressed down gently, the interlaminar fluid could be seen flowing out, the double anterior sign disappeared immediately, and the corneal edema decreased gradually within 5 days without recurrence.

**FIGURE 4:**
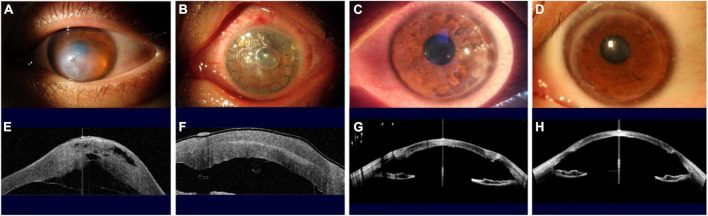
Slit-lamp photographs and anterior segment optical coherence tomography (AS–OCT) scans show the evolution of a patient with ACH treated using the novel techniques. **(A,E)** Acute onset ACH, with severe edema of the corneal stroma evident under slit-lamp examination and with a DM break and detachment observed by AS-OCT. **(B,F)** One day after the first-stage surgery, at presentation, with double layers of cornea observed on slit-lamp and AS-OCT examinations, with no corneal edema and with DM attached. **(C,G)** One week after the second-stage surgery, the corneal graft, which was the same graft used in the first-stage operation, had survived and attached to the host’s residual stroma bed with a healed DM and corneal endothelial. **(D,H)** One year after the second stage surgery, the cornea was transparent and the logMAR CDVA was 0.096.

At 6 months after DALK, the CDVA was restored to LogMAR 0.20 ± 0.11 in this group of cases, and the graft-recipient bed was well attached on observation of AS-OCT images (Figure 4). The central and paracentral corneal thickness were significantly reduced compared with pre-operative measures, and the thickness of central residual corneal stroma was 20 ± 6 μm (Table 2).

**TABLE 2:** AS-OCT results preoperatively and 6 months after the second-stage surgery.

Case	Sex	Age	Eye	logMAR CDVA Pre/Post	T_a_ (μm) Pre/Post	T_nh_ (μm) Pre/Post	T_th_ (μm) Pre/Post	T_nv_ (μm) Pre/Post	T_tv_ (μm) Pre/Post	T_r_ (μm)
1	M	18	right	1.52/0.22	1,770/631	1,230/672	1,663/660	1,023/662	1,120/657	15
2	F	19	left	2.30/0.40	2,271/654	1,543/702	1,346/698	1,106/685	923/674	25
3	M	21	right	1.52/0.13	1,880/608	1,787/648	1,530/631	1,700/629	1,305/641	28
4	M	18	left	1.70/0.10	2,082/566	1,503/585	1,556/578	1,606/581	1,692/572	24
5	M	17	left	1.85/0.28	1,621/594	1,302/624	1,140/632	1,550/612	1,321/631	21
6	F	19	left	1.30/0.22	1,592/587	1,040/602	992/610	1,201/620	985/633	11
7	M	20	right	2.00/0.09	1,874/636	1,298/700	1,660/698	1,222/673	1,320/667	18
Mean ± SD				1.74 ± 0.34/0.20 ± 0.11	1,870 ± 243/611 ± 31	1,368 ± 244/648 ± 46	1,412 ± 263/644 ± 45	1,344 ± 269/637 ± 37	1,238 ± 258/639 ± 34	20 ± 6
*t* value				13.227	14.258	8.097	8.026	6.294	5.548	
*P* value				<0.0001	<0.0001	<0.0001	<0.0001	0.001	0.001	

*Pre/Post* preoperative results and postoperative results (6 months after DALK).

## Discussion

ACH is a severe complication of keratoconus, caused by disruption and local detachment of the DM (5, 6). Due to its elasticity, DM retracts or coils when it breaks under tension, and the aqueous humor flows into the corneal stroma, causing severe corneal edema and opacity, and even leading to the formation of intrastromal clefts (pseudocysts). Classic medical treatment includes the use of hypertonic sodium chloride and cycloplegics, compressive bandaging (16, 17), and surgical treatments such as injection of inert gas (C_3_F_8_ or SF_6_) into the anterior chamber (18), corneal cross-linking, compressive sutures (19, 20), epikeratophakia or lamellar keratoplasty (21, 22). The severe corneal edema caused by large breaks in DM also limits the likelihood of a complete and successful DALK, which is now the preferred treatment for keratoconus (23).

We recognized that repairing the break in DM would allow the corneal edema to subside and the corneal stroma to heal, and could create conditions supportive of subsequent lamellar keratoplasty and even DALK. We therefore proposed this novel and effective procedure which combined epikeratophakia with intracameral air injection and DALK using the same corneal graft. Intracameral air injection is an effective therapy that can shorten the period of corneal edema (22). In the first-stage surgery, the pathological cornea was covered with an allogenic donor graft and an air bubble was inserted into the anterior chamber to push the detached DM back and adjacent to the stroma. This contributed to rapid closure of the DM wound, after which the severe edema of the recipient graft bed subsided within 1–2 days, as confirmed by the AS-OCT examination. Six months later, the DM repair was strengthened by new collagen deposits secreted by the surrounding healthy corneal endothelial cells (23), providing a solid structural foundation for the second-stage DALK surgery in the present study, including manual stromal dissection. In the 6 months of follow-up after DALK, AS-OCT examinations showed corneal (stroma plus DM) thickness and CDVA values comparable to those following DALK in keratoconus patients without ACH (24).

To our knowledge, this two-step procedure of thermokeratoplasty assisted epikeratophakia with intracameral air injection and DALK has not been reported previously in the medical literature. Currently, sustainable eye banks remain rare in developing countries, and a shortage of donor corneas is the most common problem faced by the corneal surgeon (25). The novel procedure we have proposed consists of two keratoplasties, but the same donor graft is used for both procedures, which therefore imposes no additional donor burden and technically avoids the need for a fresh donor cornea for penetrating keratoplasty with associated complications such as endothelial rejection and chronic endothelial dysfunction (26, 27). In addition, about 2 months after epikeratophakia, the donor graft epithelium had also reepithelized from the limbus of the recipient cornea. Consequently, transplanting the same donor graft in the-second stage DALK surgery also avoided the challenge of regenerating a new corneal epithelial layer, promoting rapid graft healing (28).

At 2 months after epikeratophakia, 1 eye of 1 patient in this case series showed a complication in the form of a branch-shaped neovascularization in the superficial recipient corneal stroma bed. In that patient, all corneal neovascularization was removed after thoroughly dissection during the DALK procedure, and the recipient corneal stroma bed remained transparent during subsequent follow-up.

In conclusion, we propose a novel two-stage procedure for treating ACH, which shows rapid absorption of corneal edema and DM healing and minimizes the risk of postoperative endothelial immune rejection.

## Data availability statement

The original contributions presented in this study are included in this article/supplementary material, further inquiries can be directed to the corresponding authors.

## Ethics statement

The studies involving human participants were reviewed and approved by the Medical Ethical Committee of Tongji Hospital affiliated to Tongji University. The patients/participants provided their written informed consent to participate in this study.

## Author contributions

YB and LZ: conception and review. YB and YZ: clinical operation. CL, XH, and JS: methodology, formal analysis, and writing. YB: funding acquisition. All authors contributed to the article and approved the submitted version.
